# Epidemiology and clinical management of acute diarrhoea in dogs under primary veterinary care in the UK

**DOI:** 10.1371/journal.pone.0324203

**Published:** 2025-06-11

**Authors:** Dan G. O’Neill, Lauren J. Prisk, Dave C. Brodbelt, David B. Church, Fergus Allerton

**Affiliations:** 1 Pathobiology and Population Sciences, The Royal Veterinary College, Hatfield, Herts, United Kingdom; 2 Clinical Sciences and Services, The Royal Veterinary College, Hatfield, Herts, United Kingdom; 3 Willows Veterinary Centre and Referral Service, Solihull, West Midlands, United Kingdom; Texas A&M University College Station, UNITED STATES OF AMERICA

## Abstract

**Background:**

Acute diarrhoea is a common canine veterinary presentation in the UK. This study aimed to report the incidence, demographic risk factors and clinical management for acute diarrhoea diagnosed under primary veterinary care in the UK in 2019.

**Methods:**

A cohort study design with a cross-sectional analysis was applied to anonymised VetCompass clinical data. Risk factor analysis used multivariable logistic regression modelling.

**Results:**

The analysis included a random sample of 1,835 confirmed incident acute diarrhoea cases in 2019 from an overall study population of 2,250,417 dogs. After accounting for subsampling, the estimated one-year incidence risk for acute diarrhoea in dogs overall was 8.18% (95% CI: 7.83–8.55). Of the first acute diarrhoea event in 2019 for the 1,835 cases, 1473 (80.27%) had only one physical visit for veterinary care related to the acute diarrhoea. The most common comorbid clinical signs with acute diarrhoea included vomiting (n = 812, 44.25%), reduced appetite (508, 27.68%) and lethargy (444, 24.20%). Overall, 538 (29.32%) cases were recorded as haemorrhagic diarrhoea. The most common clinical managements were probiotics (n = 1094, 59.62%), dietary management (807, 43.98%), antibiosis (701, 38.20%) and maropitant (441, 24.03%). Six breeds showed increased odds of acute diarrhoea compared with crossbred dogs: Maltese (OR 2.17, 95% CI 1.25–3.77), Miniature Poodle (OR 2.17, 95% CI 1.19–3.95), Cavapoo (OR 2.07, 95% CI 1.32–3.25), German Shepherd Dog (OR 1.69, 95% CI 1.29–2.22), Yorkshire Terrier (OR 1.51, 95% CI 1.15–1.98) and Cockapoo (OR 1.36, 95% CI 1.05–1.74). The odds of diagnosis increased in dogs aged under 3 years and dogs aged over 9 years, compared to dogs aged 4–5 years.

**Conclusions:**

This study confirms acute diarrhoea as a common clinical condition in dogs managed under primary veterinary care, with 1-in-12 dogs diagnosed each year. The identified breed predispositions suggest some genetic element to the condition. The clinical outcomes following veterinary care appear to be very positive, with over 80% of acute diarrhoea cases not receiving a second veterinary visit. However, antibiotic use remained frequent, despite years of recommendation to the contrary and raises concerns about unnecessary antibiotic therapy for this condition.

## Introduction

Acute diarrhoea is a common reason for presentation for small animal veterinary care in the UK [1–4]. The reported annual prevalence of all forms of diarrhoea of 3.8% among dogs seen in primary care practice in the UK [5] and 2.2% in the USA [6] may even underestimate the true frequency of overall diarrhoea among veterinary caseloads as some cases may have been classified under alternative diagnosis terms that subsume the diarrhoea element within wider diagnostic terms such as gastroenteritis [5]. Indeed, diarrhoea as a clinical entity is likely much more frequent in the wider dog population, given that only 37% of dogs with any form of diarrhoea are reported to receive veterinary care [7]. Owner-reported prevalence of acute diarrhoea over a two week period has been published at 14.9% in Great Britain [8] and reaching 35.1% during the same timeframe following adoption from a shelter [3].

While the clinical signs of acute diarrhoea are typically self-limiting [9], with 78% resolution reported within 2 days [3], antibiotic treatment is reported as often used in dogs under veterinary care with the aim of reducing the severity or duration of the diarrhoea [10,11]. Antibiotic prescription has been recorded in 28–70% of canine diarrhoea cases [10,12–14]. However, a recent meta-analysis indicates that antibiotic therapy is unnecessary in the majority of acute diarrhoea cases [15], with a target trial emulation of a randomised controlled trial (RCT) using observational veterinary clinical data in the UK also revealing no clinical value from routine antimicrobial therapy of uncomplicated acute diarrhoea in dogs [16]. Antimicrobial stewardship guidelines consistently advise against routine antibiotic use to manage acute and chronic diarrhoea [17]. A recent European multidisciplinary panel has examined the published evidence and incorporated the opinions of general veterinary practitioners and dog owners. The panel strongly recommend and with high certainty of evidence that antimicrobials should not be used to treat dogs with acute haemorrhagic or non-haemorrhagic diarrhoea that present with mild (i.e., dogs in good general condition, with no signs of dehydration or systemic illness) or moderate (i.e., dogs with impaired general condition and varying degrees of dehydration/hypovolemia. Dogs may have signs of systemic disease related to the deficit of body fluids that will resolve with adequate fluid therapy) disease [18].

A recent UK study found that 70.8% of antibiotic prescriptions were not consistent with the ‘BSAVA/SAMSoc Guide to Responsible Use of Antibiotics: PROTECT ME’ Guidelines on antibiotic use for diarrhoea [19,20]. In-depth interviews with pet owners and veterinarians have identified a tension between appropriate antibiotic stewardship by the veterinarians and the perceived satisfaction with the clinical care by the pet owner [21]. Veterinary antibiotic prescribing on a ‘just in case’ basis seems to remain relatively common, often driven by veterinary concerns about perceived client anxiety for the health and welfare of their pet even though many pet owners self-declare a high receptiveness to non-antibiotic approaches [22,23]. This apparent veterinarian-owner discordance highlights a major challenge facing veterinarians in the consulting room and be contributing to unnecessary and excessive antibiotic prescription. Access to better evidence-based data derived from a representative primary care population on antibiotic usage for diarrhoea might help to mitigate some of this unnecessary prescribing behaviour.

The ‘BSAVA/SAMSoc Guide to Responsible Use of Antibiotics: PROTECT ME’ Guidelines on antibiotic use for diarrhoea stratify animals based on clinical status alone and do not make accommodations for potential influencing factors such as age, breed or sex [20]. This lack of granularity may reflect knowledge gaps around the role of signalment to predict diarrhoea risk and outcomes rather than a true belief that signalment does not matter. Many previous studies on acute diarrhoea either did not describe the population characteristics in detail, were limited to single breeds or reported on specific conditions such as acute haemorrhagic syndrome, often in a referral institution. A greater understanding of the demography of dogs with acute diarrhoea could support veterinarians to select better targeted therapeutic and preventative strategies.

Using anonymised veterinary clinical data from the VetCompass Programme [24], this study aimed to report the incidence, demographic risk factors and clinical management for acute diarrhoea diagnosed under primary veterinary care in the UK in 2019. Breed effects were given particular focus as a risk factor. These results could assist veterinary practitioners, welfare scientists, breeders, and owners with a stronger evidence base to predict, prevent and better manage acute diarrhoea in dogs.

## Methods

The study population included all dogs under primary veterinary care at clinics participating in the VetCompass Programme during 2019. Dogs under veterinary care were defined as those with ≥ 1 electronic health record (EHR) (free-text clinical note, treatment or bodyweight) recorded during 2019. VetCompass collates de-identified EHR data from primary-care veterinary practices in the UK for epidemiological research [24]. Data fields for each animal included fixed values for species, breed, date of birth, sex and neuter status along with date-specific information on free-form text clinical notes, bodyweight and treatment.

A cohort study design with a cross-sectional analysis was used to estimate the one-year (2019) incidence risk of acute diarrhoea and to explore associations between demographic risk factors and acute diarrhoea. Based on prior evidence for 3.81% annual prevalence of diarrhoea diagnosed under primary veterinary care in the UK [5], power calculation estimated that a study sample of 22,462 dogs was needed to estimate incidence risk for a disorder that occurred in 3.81% of dogs with 0.25% acceptable margin of error at a 95% confidence level from a national UK population of 8 million dogs [25,26]. Ethics approval was obtained from the RVC Ethics and Welfare Committee (reference SR2018−1652).

The case definition for an acute diarrhoea case required evidence in the clinical records for a final diagnosis of at least one incident event of uncomplicated acute diarrhoea (or synonym) at any date from Jan 1, 2019 to Dec 31, 2019. Uncomplicated acute diarrhoea described diarrhoea events that were not recorded within a broader formal diagnosis of another condition such as confirmed poisoning, infection or systemic disease. An incident acute diarrhoea event required no evidence for diarrhoea existing during the preceding 30 days. The clinical diagnosis of these cases relied on the clinical acumen of the veterinarians and the confirmation of meeting the case definition relied on the information recorded in the clinical records. Case-finding involved initial screening of all 2,250,417 study dogs for candidate acute diarrhoea cases by searching the clinical free-text field and treatment free-text field from 1^st^ January 2019–31^st^ December 2019 using a list of search terms. The clinical free-text field was searched using d + , prokolin, probind, ‘egg count’, protexin, diarr*, gastroenteritis, enteritis, gastritis, loose and stools. The treatment field was searched using prokolin, probind, protexin, glutalyte, buscopan, loperamide and kaolin. The clinical notes of a random sample of candidate acute diarrhoea cases were manually reviewed to evaluate for case inclusion. Additional information was manually extracted for confirmed acute diarrhoea cases on the date of diagnosis, number of physical veterinary visits for the clinical event, clinical signs on presentation, diagnostic approaches and treatments, and the stated suspected trigger for the acute diarrhoea event. All dogs that were not screened as candidate cases were included as non-cases in the risk factor analysis.

Breed descriptive information entered by the participating practices was cleaned and mapped to a VetCompass breed list derived and extended from the VeNom Coding breed list that included both recognised purebred breeds and also designer crossbreed breed terms [27]. A *breed purity* variable categorised all dogs of recognisable breeds as ‘purebred’, dogs with contrived names generated from two or more purebred breed terms as ‘designer crossbreed’ crossbreds (purposely bred crossbreeds), and dogs recorded as mixes of breeds but without a contrived name as ‘crossbred’ [28]. A *breed* variable included individual pure breeds and designer hybrids represented by over 10,000 dogs in the overall study population or with ≥ 10 acute diarrhoea cases, along with a grouping of all remaining breeds and also a grouping of general crossbred dogs. A skull shape variable categorised dogs based on their breed information as dolichocephalic, mesocephalic, brachycephalic or uncategorised [29]. A *Kennel Club breed group* variable classified breeds recognised by the UK Kennel Club into their relevant breed groups (Gundog, Hound, Pastoral, Terrier, Toy, Utility and Working) and all remaining types were classified as non-Kennel Club recognised [28].

Consistent with methods previously used [30], neuter status was defined at the final available EHR. Adult bodyweight was defined as the median of all bodyweight (kg) values recorded for each dog after reaching 18 months old and was categorised as: < 10.0, 10.0 to < 15.0, 15.0 to < 20.0, 20.0 to < 25.0, 25.0 to < 30.0, 30.0 to < 40.0, 40.0 to < 50.0, 50.0 to < 60.0 and ≥ 60.0. Age (years) was defined for each dog on December 31, 2019 and was categorised in one-year bands to 24 years.

Following internal validity checking and data cleaning in Excel (Microsoft Office Excel 2013, Microsoft Corp.), analyses were conducted using Stata Version 16 (Stata Corporation). Annual incidence risk with 95% confidence intervals (CI) described the probability of at least one incident event of acute diarrhoea at any point during 2019. Because the sampling design involved verification of a subset of candidate cases, the incidence risk and 95% CI were based on the number of confirmed cases from the sampling proportion of the overall study population. The CI estimates were derived from standard errors, based on approximation to the binomial distribution [31]. Results for clinical management were reported descriptively. Univariable associations between categorical variables were assessed using the chi^2^ test. Normality of distributions for continuous variables was assessed using histograms, with non-normally distributed continuous variables reported using median, interquartile range (IQR) and range. Risk factor analysis used binary logistic regression modelling to evaluate univariable associations between risk factors (*breed, skull shape, breed purity, Kennel Club recognised breed, Kennel Club breed group, adult bodyweight, age, sex and neuter*) and incident acute diarrhoea during 2019. Because breed was a factor of primary interest for the study, variables that derived from the breed term and therefore were highly correlated with breed (*skull shape, breed purity, Kennel Club recognised breed* and *Kennel Club breed group*) were excluded from initial breed multivariable modelling. Instead, each of these variables individually replaced the *breed* variable in the main breed-focused model to evaluate their effects after taking account of the other variables. *Adult bodyweight* (a defining characteristic of individual breeds) also replaced breed in the final breed-focused model. Risk factors with liberal associations in univariable modelling (*P *< 0.2) were taken forward for multivariable evaluation. Model development used manual backwards stepwise elimination. Pair-wise interaction effects were evaluated for the final model variables [32]. The area under the ROC curve and McKelvey & Zavoina Pseudo-R² were used to evaluate the quality of the model fit [32,33]. Statistical significance was set at *P *< 0.05.

## Results

### Incidence risk

**Fig 1:**
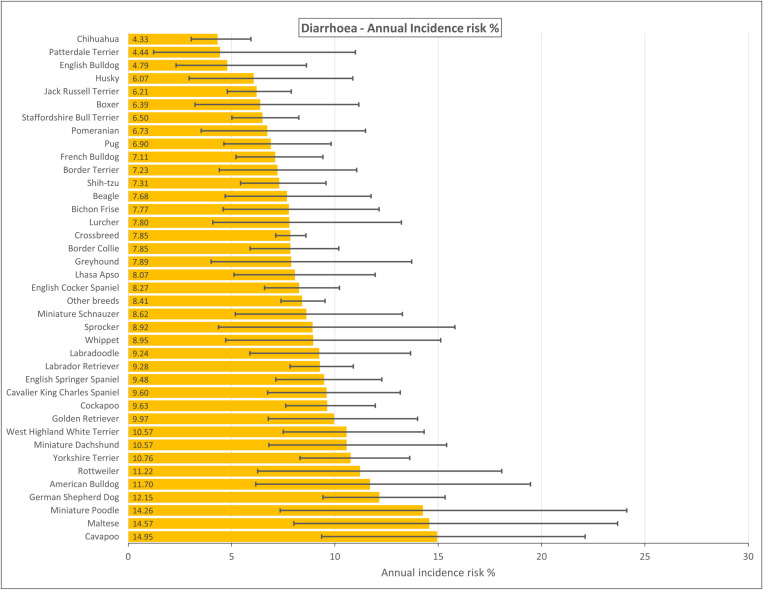
Annual incidence risk (%) of acute diarrhoea in dog breeds under primary veterinary care in the VetCompass Programme in the UK in 2019. The horizontal bars represent 95% confidence intervals.

Text searches of an overall study population of 2,250,417 dogs under primary veterinary care in 2019 yielded 808,241 candidate acute diarrhoea cases with some indication of diarrhoea in 2019. Manual checking of a random sample of 8,053/808,241 (1.00% sampling proportion) candidate cases identified 1,835 confirmed incident acute diarrhoea cases during 2019 from a notional 22,422 study sample (i.e., 1.00% sampling proportion of study population). After accounting for the subsampling protocol, the estimated one-year incidence risk for acute diarrhoea in dogs overall was 8.18% (95% CI: 7.83–8.55). Breeds with the highest annual incidence risk for acute diarrhoea were Cavapoo (incidence risk 14.95%, 95% CI 9.36–22.11), Maltese (14.57%, 95% CI 8.01–23.68), Miniature Poodle (14.26%, 95% CI 7.35–24.13) and German Shepherd Dog (12.15%, 9.42–15.33) (Fig 1). Of the 1,835 acute diarrhoea cases, the most presented breeds were Crossbreed (n = 417, 22.72%), Labrador Retriever (n = 135, 7.36%), English Cocker Spaniel (n = 78, 4.25%) and Cockapoo (n = 73, 3.98%).

Of the acute diarrhoea cases with data available for that variable, 1,267 (69.20%) were purebred, 879 (48.16%) were female and 822 (45.04%) were neutered. Dogs with acute diarrhoea had a median adult bodyweight of 13.80 kg (IQR: 8.70–25.00, range 1.90–67.50) and median age was 4.93 years (IQR: 1.63–9.88, range 0.01–21.74).

Of the dogs that were not acute diarrhoea cases with data available on the variable, 990,335 (69.17%) were purebred and 688,458 (48.23%) were female, 601,570 (42.14%) were neutered. The median adult bodyweight for non-cases was 13.60 kg (IQR: 8.30–24.30, range 0.38–106.00) and the median age was 5.26 years (IQR: 2.40–8.81, range 0.00–24.98). The breeds most presented as non-cases were Crossbreed (350,238, 24.29%), Labrador Retriever (95,938, 6.65%), Jack Russell Terrier (67,307, 4.67%) and Staffordshire Bull Terrier (62,488, 4.33%) (Table 1).

**Table 1:** Descriptive and univariable logistic regression results for breed as a risk factor for incident cases of acute diarrhoea during 2019 in dogs under primary veterinary care in the VetCompass™ Programme in the UK. Column percentages shown in brackets. P-values < 0.050 are bolded. *CI confidence interval.

Breed	Case No. (%)	Non-case No. (%)	Odds Ratio	95% CI	Category *P*-value	Variable *P*-value
Crossbreed	417 (22.72)	350238 (24.29)	Base	–		< 0.001
Miniature Poodle	11 (0.60)	4016 (0.28)	2.30	1.26-4.19	**0.006**	
Maltese	13 (0.71)	5159 (0.36)	2.12	1.22-3.68	**0.008**	
Cavapoo	20 (1.09)	8435 (0.58)	1.99	1.27-3.12	**0.003**	
American Bulldog	12 (0.65)	5856 (0.41)	1.72	0.97-3.06	0.064	
German Shepherd Dog	61 (3.32)	30320 (2.1)	1.69	1.29-2.21	**< 0.001**	
Golden Retriever	29 (1.58)	16155 (1.12)	1.51	1.03-2.20	**0.033**	
Yorkshire Terrier	60 (3.27)	34054 (2.36)	1.48	1.13-1.94	**0.005**	
Labradoodle	22 (1.2)	13100 (0.91)	1.41	0.92-2.17	0.116	
Cockapoo	73 (3.98)	44037 (3.05)	1.39	1.09-1.79	**0.009**	
West Highland White Terrier	36 (1.96)	21833 (1.51)	1.38	0.98-1.95	0.061	
Cavalier King Charles Spaniel	34 (1.85)	20916 (1.45)	1.37	0.96-1.94	0.081	
Sprocker	10 (0.54)	6179 (0.43)	1.36	0.73-2.55	0.338	
Rottweiler	14 (0.76)	8653 (0.6)	1.36	0.80-2.32	0.259	
English Springer Spaniel	51 (2.78)	32633 (2.26)	1.31	0.98-1.76	0.067	
Beagle	19 (1.04)	12623 (0.88)	1.26	0.80-2.00	0.318	
Miniature Dachshund	23 (1.25)	15361 (1.07)	1.26	0.83-1.91	0.285	
Whippet	12 (0.65)	8099 (0.56)	1.24	0.70-2.21	0.455	
Miniature Schnauzer	18 (0.98)	12336 (0.86)	1.23	0.76-1.97	0.399	
Labrador Retriever	135 (7.36)	95938 (6.65)	1.18	0.97-1.44	0.092	
Greyhound	11 (0.6)	7925 (0.55)	1.17	0.64-2.12	0.616	
Lhasa Apso	22 (1.2)	15997 (1.11)	1.16	0.75-1.77	0.510	
English Cocker Spaniel	78 (4.25)	59973 (4.16)	1.09	0.86-1.39	0.474	
Other	228 (12.43)	176895 (12.27)	1.08	0.92-1.27	0.336	
Border Collie	51 (2.78)	40592 (2.81)	1.06	0.79-1.41	0.717	
Pomeranian	12 (0.65)	9597 (0.67)	1.05	0.59-1.86	0.867	
Border Terrier	19 (1.04)	15211 (1.05)	1.05	0.66-1.66	0.838	
Lurcher	12 (0.65)	10519 (0.73)	0.96	0.54-1.70	0.884	
Shih Tzu	48 (2.62)	43048 (2.98)	0.94	0.69-1.26	0.667	
Bichon Frise	17 (0.93)	15841 (1.1)	0.90	0.55-1.46	0.675	
Pug	28 (1.53)	27026 (1.87)	0.87	0.59-1.28	0.476	
French Bulldog	44 (2.4)	42954 (2.98)	0.86	0.63-1.17	0.343	
Boxer	11 (0.6)	10834 (0.75)	0.85	0.47-1.55	0.602	
Staffordshire Bull Terrier	62 (3.38)	62488 (4.33)	0.83	0.64-1.09	0.181	
Jack Russell Terrier	62 (3.38)	67307 (4.67)	0.77	0.59-1.01	0.060	
Husky	10 (0.54)	11545 (0.8)	0.73	0.39-1.36	0.320	
English Bulldog	10 (0.54)	15304 (1.06)	0.55	0.29-1.03	0.061	
Chihuahua	36 (1.96)	56005 (3.88)	0.54	0.38-0.76	**< 0.001**	
Patterdale Terrier	4 (0.22)	7174 (0.5)	0.47	0.17-1.25	0.131	

### Clinical

Among the 1,835 incident acute diarrhoea cases, 1,689 (92.04%) had just one event of acute diarrhoea recorded during 2019, with 2 events for 129 (7.03%), 3 events for 13 (0.71%), 4 events for 3 (0.16%) and 5 events for 1 (0.05%). To avoid biasing towards effects from dogs with multiple events, the clinical results from here are reported based on only the earliest acute diarrhoea event in 2019 for each case. The duration of the acute diarrhoea prior to seeking veterinary care was stated in the clinical notes for 1508/1835 (82.18%) cases. Of these, the duration was less than 24 hours for 464 (30.77%), 24 to < 48 hours for 364 (24.14%), 48 to < 96 hours for 400 (26.53%), 4 to < 7 days for 169 (11.21%), 1 to < 2 weeks for 79 (5.24%) and > 2 weeks for 32 (2.12%). For the first acute diarrhoea event for the 1,835 cases, 1473 (80.27%) had one physical visit for veterinary care related to the acute diarrhoea, with 202 (11.01%) having two physical visits, 62 (3.38%) having three physical visits and 19 (1.04%) having four physical visits. Seventy-nine (4.31%) cases were clinically managed remotely.

Diarrhoea was the only clinical signs recorded for 604 (32.92%) acute diarrhoea cases. The most common other clinical signs associated with the acute diarrhoea recorded on the day of first veterinary presentation included vomiting (n = 812, 44.25%), reduced appetite (508, 27.68%), lethargy (444, 24.20%), abdominal pain/discomfort (246, 13.41%), pyrexia (160, 8.72%) and dehydration (143, 7.79%). (S1 File).

Information on the physical nature of the diarrhoea was recorded for 1241 (67.63%) acute diarrhoea cases. Overall, 538 cases were recorded as haemorrhagic (29.32% of all cases, 37.86% of cases with some description provided). No laboratory diagnostic testing was conducted on the first day of veterinary presentation for 1511 (82.34%) cases. The most common laboratory diagnostic tests conducted on the first day of veterinary presentation were complete haematology + /- biochemistry (n = 202, 11.01%), faecal analysis (including antigen testing, egg counts or bacterial culture and sensitivity) (64, 3.49%), abdominal imaging (64, 3.49%) and cPLI Snap test (IDEXX UK) (46, 2.51%) (S2 File). Haemorrhagic diarrhoea cases (101/538, 18.77%) were not more likely to have at least one laboratory diagnostic test conducted on the first day of veterinary presentation compared with non-haemorrhagic diarrhoea cases (223/1297, 17.19%) (chi^2^ p-value 0.419). Acute diarrhoea cases that underwent diagnostic testing were more likely to receive antibiotics (145/324, 44.75%) than cases that did not undergo diagnostic testing (556/1511, 36.80%) (chi^2^ p-value 0.007).

The most common treatment or clinical management for acute diarrhoea cases were probiotics (n = 1094, 59.62%), dietary management (807, 43.98%), antibiosis (701, 38.20%) and maropitant (441, 24.03%). There were 132 (7.19%) of the cases hospitalised as part of the clinical management (S3 File). Haemorrhagic diarrhoea cases (315/538, 58.55%) were significantly more likely to be prescribed antibiotics on the first day of veterinary presentation compared with non-haemorrhagic diarrhoea cases (386/1297, 29.76%) (chi^2^ p-value < 0.001). The most prescribed antibiotics and their routes of administration on first presentation of the 701 cases treated with antibiotics were oral metronidazole (n = 457, 65.19%), parenteral amoxicillin (159, 22.68%) and oral amoxicillin (116, 16.55%) (S4 File). No information on a suspected trigger for the acute diarrhoea event was suggested in the clinical records for 736 (40.11%) of the acute diarrhoea events. The most commonly recorded suspected trigger or explanation for the acute diarrhoea were scavenging/dietary indiscretion (n = 175, 9.54%), gastroenteritis (149, 8.12%), colitis (85, 4.63%), reaction to NSAID therapy (85, 4.63%) and dietary change (84, 4.58%) (S5 File).

### Risk factors

Seven study variables (breed, breed purity, Kennel Club breed group, skull conformation, age (years), neuter status and veterinary group) were liberally associated with acute diarrhoea in univariable logistic regression modelling and were evaluated using multivariable logistic regression modelling. Three variables were not associated with acute diarrhoea at a univariable level and were not considered further: Kennel Club recognised breed, adult (> 18 months) bodyweight (kg), and sex (Tables 1–3). The final breed-focused multivariable model retained four risk factors: breed, age (years), neuter status and veterinary group (Table 4). No biologically significant interactions were identified. McKelvey & Zavoina’s R^2^ value of 0.0673 showed that 6.73% of the total variance was explained by the risk factors in the model. The final model showed moderate discrimination (area under the ROC curve: 0.652).

**Table 2:** Descriptive and univariable logistic regression results for breed-derived risk factors for incident cases of acute diarrhoea during 2019 in dogs under primary veterinary care in the VetCompass™ Programme in the UK. Column percentages shown in brackets. P-values < 0.050 are bolded. *CI confidence interval.

Variable	Category	Case No. (%)	Non-case No. (%)	Odds ratio	95% CI*	Category *P*-value	Variable *P*-value
Breed purity	General crossbred	417 (22.77)	350,238 (24.46)	Base			0.009
	Designer crossbreed	147 (8.03)	91,159 (6.37)	1.35	1.12-1.63	**0.002**	
	Purebred	1,267 (69.20)	990,335 (69.17)	1.07	0.96-1.20	0.203	
Kennel Club Recognised Breed	Not recognised	592 (32.33)	468,366 (32.71)	Base			0.728
	Recognised	1,239 (67.67)	963,366 (67.29)	1.02	0.92-1.12	0.728	
Kennel Club Breed Group	Not Kennel Club recognised breed	592 (32.33)	468,366 (32.71)	1.00			< 0.001
	Gundog	341 (18.62)	229,931 (16.06)	1.17	1.03-1.34	**0.019**	
	Hound	89 (4.86)	61,074 (4.27)	1.15	0.92-1.44	0.211	
	Pastoral	135 (7.37)	82,133 (5.74)	1.30	1.08-1.57	**0.006**	
	Terrier	215 (11.74)	189,963 (13.27)	0.90	0.77-1.05	0.166	
	Toy	216 (11.80)	181,685 (12.69)	0.94	0.80-1.10	0.441	
	Utility	186 (10.16)	168,588 (11.78)	0.87	0.74-1.03	0.106	
	Working	57 (3.11)	49,992 (3.49)	0.90	0.69-1.18	0.458	
Skull conformation	Mesocephalic	811 (64.01)	614,690 (62.07)	Base			< 0.001
	Brachycephalic	263 (20.76)	258,218 (26.07)	0.77	0.67-0.89	**< 0.001**	
	Dolichocephalic	193 (15.23)	117,427 (11.86)	1.25	1.06-1.46	**0.006**	

**Table 3:** Descriptive and univariable logistic regression results for non-breed-related demographic risk factors evaluated for incident cases of acute diarrhoea during 2019 in dogs under primary veterinary care in the VetCompass™ Programme in the UK. Column percentages shown in brackets. P-values < 0.050 are bolded. *CI confidence interval.

Variable	Category	Case No. (%)	Non-case No. (%)	Odds ratio	95% CI*	Category *P*-value	Variable *P*-value
Adult (> 18 months) bodyweight (kg)	<10 kg	458 (33.68)	332,273 (35.28)	1.00			0.412
	10.0- < 15.0 kg	268 (19.71)	173,640 (18.44)	1.12	0.96-1.30	0.142	
	15.0- < 20.0 kg	151 (11.10)	1145,28 (12.16)	0.96	0.80-1.15	0.636	
	20.0- < 25.0 kg	141 (10.37)	98,145 (10.42)	1.04	0.86-1.26	0.668	
	25.0- < 30.0 kg	145 (10.66)	85,697 (9.10)	1.23	1.02-1.48	**0.032**	
	30.0- < 40.0 kg	151 (11.10)	107,114 (11.37)	1.02	0.85-1.23	0.811	
	40.0- < 50.0 kg	38 (2.79)	23,354 (2.48)	1.18	0.85-1.64	0.326	
	50.0- < 60.0 kg	6 (0.44)	4,774 (0.51)	0.91	0.41-2.04	0.822	
	> or = 60 kg	2 (0.15)	2,279 (0.24)	0.64	0.16-2.55	0.524	
Age (years)	<1.0	224 (12.24)	141,910 (9.94)	2.05	1.61-2.61	**< 0.001**	< 0.001
	1.0- < 2.0	316 (17.27)	155,003 (10.86)	2.65	2.11-3.34	**< 0.001**	
	2.0- < 3.0	183 (10.00)	141,248 (9.90)	1.69	1.31-2.16	**< 0.001**	
	3.0- < 4.0	104 (5.68)	124,543 (8.73)	1.09	0.82-1.44	0.559	
	4.0- < 5.0	94 (5.14)	122,345 (8.57)	Base			
	5.0- < 6.0	89 (4.86)	111,822 (7.84)	1.04	0.78-1.38	0.812	
	6.0- < 7.0	100 (5.46)	104,517 (7.32)	1.25	0.94-1.65	0.127	
	7.0- < 8.0	90 (4.92)	95,995 (6.72)	1.22	0.91-1.63	0.177	
	8.0- < 9.0	95 (5.19)	87,951 (6.16)	1.41	1.06-1.87	**0.019**	
	9.0- < 10.0	85 (4.64)	77,027 (5.40)	1.44	1.07-1.93	**0.016**	
	10.0- < 11.0	88 (4.81)	67,475 (4.73)	1.70	1.27-2.27	**< 0.001**	
	11.0- < 12.0	96 (5.25)	56,729 (3.98)	2.20	1.66-2.93	**< 0.001**	
	12.0- < 13.0	84 (4.59)	46,749 (3.28)	2.34	1.74-3.14	**< 0.001**	
	13.0- < 14.0	70 (3.83)	35,857 (2.51)	2.54	1.86-3.46	**< 0.001**	
	14.0- < 15.0	55 (3.01)	25,930 (1.82)	2.76	1.98-3.85	**< 0.001**	
	15.0- < 16.0	28 (1.53)	16,250 (1.14)	2.24	1.47-3.42	**< 0.001**	
	16.0- < 17.0	18 (0.98)	8,674 (0.61)	2.70	1.63-4.47	**< 0.001**	
	17.0- < 18.0	11 (0.60)	7,066 (0.50)	2.03	1.08-3.79	**0.027**	
Sex	Female	879 (48.16)	688,458 (48.23)	Base			0.955
	Male	946 (51.84)	738,976 (51.77)	1.00	0.91-1.10	0.955	
Neuter status	Entire	1,003 (54.96)	825,864 (57.86)	Base			0.013
	Neutered	822 (45.04)	601,570 (42.14)	1.13	1.03-1.23	**0.012**	
Veterinary Group	A	576 (0.11)	534,408 (99.89)	Base			
	B	2 (0.16)	1,274 (99.84)	1.46	0.36-5.84	0.596	< 0.001
	C	579 (0.17)	342,796 (99.83)	1.57	1.40-1.76	**< 0.001**	
	D	33 (0.15)	21,852 (99.85)	1.40	0.99-1.99	0.060	
	E	311 (0.13)	232,289 (99.87)	1.24	1.08-1.43	**0.002**	
	F	334 (0.11)	309,557 (99.89)	1.00	0.87-1.15	0.988	

**Table 4:** Breed-centered model using multivariable logistic regression results for risk factors for incident cases of acute diarrhoea during 2019 in dogs under primary veterinary care in the VetCompass™ Programme in the UK. Column percentages shown in brackets. P-values < 0.050 are bolded. *CI confidence interval.

Risk factor	Category	Odds ratio	95% CI*	Category *P*-value	Variable *P*-value
Breed	Crossbreed	Base	~		< 0.001
	Maltese	2.17	1.25-3.77	**0.006**	
	Miniature Poodle	2.17	1.19-3.95	**0.012**	
	Cavapoo	2.07	1.32-3.25	**0.002**	
	German Shepherd Dog	1.69	1.29-2.22	**< 0.001**	
	American Bulldog	1.64	0.92-2.91	0.093	
	Yorkshire Terrier	1.51	1.15-1.98	**0.003**	
	Sprocker	1.51	0.80-2.83	0.201	
	Golden Retriever	1.41	0.97-2.06	0.072	
	Labradoodle	1.41	0.92-2.16	0.118	
	Rottweiler	1.39	0.82-2.38	0.223	
	Cavalier King Charles Spaniel	1.37	0.97-1.95	0.076	
	Cockapoo	1.36	1.05-1.74	**0.018**	
	Beagle	1.30	0.82-2.07	0.259	
	West Highland White Terrier	1.28	0.91-1.81	0.155	
	English Springer Spaniel	1.27	0.95-1.70	0.113	
	Greyhound	1.26	0.69-2.30	0.446	
	Miniature Schnauzer	1.23	0.77-1.97	0.392	
	Whippet	1.22	0.69-2.17	0.494	
	Lhasa Apso	1.18	0.77-1.81	0.450	
	Miniature Dachshund	1.18	0.77-1.79	0.447	
	Labrador Retriever	1.13	0.93-1.38	0.206	
	English Cocker Spaniel	1.07	0.84-1.36	0.582	
	Pomeranian	1.07	0.60-1.90	0.819	
	Other	1.06	0.90-1.25	0.461	
	Border Collie	0.99	0.74-1.33	0.947	
	Shih Tzu	0.97	0.72-1.31	0.867	
	Border Terrier	0.97	0.61-1.54	0.910	
	Bichon Frise	0.95	0.59-1.55	0.849	
	Lurcher	0.91	0.51-1.62	0.756	
	Pug	0.89	0.60-1.31	0.540	
	Boxer	0.87	0.48-1.59	0.656	
	Staffordshire Bull Terrier	0.83	0.63-1.08	0.164	
	French Bulldog	0.79	0.58-1.09	0.155	
	Husky	0.77	0.41-1.44	0.414	
	Jack Russell Terrier	0.72	0.55-0.94	**0.015**	
	Chihuahua	0.59	0.42-0.83	**0.002**	
	English Bulldog	0.55	0.29-1.03	0.062	
	Patterdale Terrier	0.45	0.17-1.20	0.110	
Age (years)	<1.0	2.42	1.89-3.09	**< 0.001**	< 0.001
	1.0- < 2.0	2.95	2.33-3.73	**< 0.001**	
	2.0- < 3.0	1.77	1.38-2.28	**< 0.001**	
	3.0- < 4.0	1.10	0.83-1.45	0.511	
	4.0- < 5.0	Base	~		
	5.0- < 6.0	1.01	0.75-1.35	0.951	
	6.0- < 7.0	1.19	0.90-1.58	0.229	
	7.0- < 8.0	1.16	0.86-1.54	0.330	
	8.0- < 9.0	1.33	1.00-1.76	0.054	
	9.0- < 10.0	1.35	1.01-1.81	**0.046**	
	10.0- < 11.0	1.59	1.19-2.13	**0.002**	
	11.0- < 12.0	2.04	1.53-2.72	**< 0.001**	
	12.0- < 13.0	2.16	1.60-2.90	**< 0.001**	
	13.0- < 14.0	2.36	1.72-3.22	**< 0.001**	
	14.0- < 15.0	2.57	1.84-3.60	**< 0.001**	
	15.0- < 16.0	2.11	1.38-3.22	**0.001**	
	16.0- < 17.0	2.57	1.55-4.27	**< 0.001**	
	17.0- < 18.0	1.99	1.06-3.72	**0.032**	
Neuter status	Entire	Base	~		< 0.001
	Neutered	1.25	1.12-1.40	**< 0.001**	
Veterinary Group	A	Base	~		
	B	1.45	0.36-5.84	0.598	< 0.001
	C	1.60	1.42-1.80	**< 0.001**	
	D	1.33	0.93-1.91	0.115	
	E	1.20	1.04-1.38	**0.012**	
	F	1.05	0.92-1.21	0.444	

After accounting for the effects of the other variables evaluated in the multivariable modelling, six breeds showed increased odds of acute diarrhoea compared with crossbred dogs: Maltese (OR 2.17, 95% CI 1.25–3.77), Miniature Poodle (OR 2.17, 95% CI 1.19–3.95), Cavapoo (OR 2.07, 95% CI 1.32–3.25), German Shepherd Dog (OR 1.69, 95% CI 1.29–2.22), Yorkshire Terrier (OR 1.51, 95% CI 1.15–1.98) and Cockapoo (OR 1.36, 95% CI 1.05–1.74). Two breeds showed reduced odds of acute diarrhoea compared with crossbreds: Jack Russell Terrier (OR 0.72, 95% CI 0.55–0.94, p-value 0.015) and Chihuahua (OR 0.59, 95% CI 0.42–0.83, p-value 0.002). The odds of diagnosis with acute diarrhoea were strongly age-related, rising in dogs aged under 3 years and dogs aged over 9 years compared to dogs aged 4–5 years. Neutered dogs showed 1.25 times higher odds of diagnosis with acute diarrhoea than entire dogs. The veterinary group attended was retained in the multivariable modelling to account for some residual confounding effects (Table 4).

As described in the methods, breed-derived variables were introduced individually to replace *breed* in the final breed-focused model. Compared with crossbred dogs, designer crossbreed dogs had 1.34 times higher odds (95% CI 1.11–1.62, p-value 0.002) of acute diarrhoea. The pastoral Kennel Club breed group showed 1.26 times higher odds (95% CI 1.05–1.52, p-value 0.015) compared to breeds not recognised by the Kennel Club. Compared with breeds with mesocephalic skull conformation, breeds with brachycephalic skull conformation (OR 0.80, 95% CI 0.70–0.92, p-value 0.002) had reduced odds of acute diarrhoea and breeds with a dolichocephalic skull conformation had increased odds (OR 1.25, 95% CI 1.07–1.46, p-value 0.006) (Table 5).

**Table 5:** Variables that replaced breed in multivariable logistic regression modelling for risk factors for incident cases of acute diarrhoea during 2019 in dogs under primary veterinary care in the VetCompass™ Programme in the UK. Column percentages shown in brackets. P-values < 0.050 are bolded. *CI confidence interval.

Risk factor	Category	Odds ratio	95% CI*	Category *P*-value	Variable *P*-value
Breed purity	General crossbred	Base	~		< 0.001
	Designer crossbreed	1.34	1.11-1.62	**0.002**	
	Purebred	1.05	0.94-1.18	0.363	
Kennel Club Breed Group	Not Kennel Club recognised breed	Base	~		< 0.001
	Gundog	1.13	0.99-1.29	0.070	
	Hound	1.14	0.91-1.43	0.248	
	Pastoral	1.26	1.05-1.52	**0.015**	
	Terrier	0.86	0.73-1.01	0.058	
	Toy	0.98	0.84-1.15	0.832	
	Utility	0.87	0.73-1.02	0.091	
	Working	0.92	0.70-1.21	0.552	
Skull conformation	Mesocephalic	Base	~		< 0.001
	Brachycephalic	0.80	0.70-0.92	**0.002**	
	Dolichocephalic	1.25	1.07-1.46	**0.006**	

## Discussion

The high frequency of acute diarrhoea in the current study population that showed a one-year incidence risk of 8.18% underlines the welfare and workflow importance of this clinical sign for veterinary professionals working in primary practice. The majority of acute diarrhoea cases in the current study fit conventional definitions that are widely accepted for acute diarrhoea, with 92% of the current cases being single presentation and 98% lasting under 14 days from presentation [34]. However, despite this evident magnitude of the disease burden posed by canine acute diarrhoea, the body of published literature around this presentation in primary practice remains sparse and has often focused on specific but rarer variants such as acute haemorrhagic diarrhoea syndrome (AHDS) [35,36] or canine parvovirus [37]. The current paper aimed to address this critical data gap to fulfil this key welfare opportunity.

The 8.18% incidence of dogs reported here with acute diarrhoea during 2019 is substantially greater than the 3.81% prevalence of overall diarrhoea (i.e., both acute and chronic) recorded previously for dogs under veterinary care in 2016 [5]. For canine acute uncomplicated diarrhoea where, by definition, disease duration is short, incidence and prevalence are likely to report very similar results, so this distinction would not explain these differing values over time [38]. It is possible that these differing results do reflect true increased care-seeking by pet owners or even effects from emergence of novel enteropathogens, e.g., a vomiting outbreak identified in early 2020 and associated with canine enteric coronavirus included diarrhoea in 50% of cases but could already have been affecting dogs during 2019 [39]. However, the most likely explanation is that these differing results over time reflect differing methods used to assign diagnostic codes for each of the two studies, with the current study including all cases that showed uncomplicated diarrhoea as a clinical sign whereas the 2016 study included only the subset of those uncomplicated diarrhoea cases where acute diarrhoea was recorded as the primary diagnosis and excluded acute diarrhoea events where a primary biomedical diagnosis such as gastroenteritis was recorded or where a co-morbid clinical sign such as vomiting was recorded as the primary diagnosis term. This distinction highlights the importance of diligence to the study methods when veterinary professionals are interpreting results as part of good evidence based veterinary medicine [40].

There is currently increasing interest in gaining a better understanding of the health and welfare of designer crossbreed dogs that represent many novel dog breeds rapidly rising in ownership [41]. Designer crossbreed breeds are defined as novel breeds generated as first-generation or later-generation crosses between two or more conventionally recognised pure breeds [42]. An existence of hybrid vigour in dogs would anticipate lower disease risk among cross breed animals compared to pure breed animals so a higher incidence of acute diarrhoea in designer crossbred dogs compared to either general crossbreds or to purebred dogs would be the inverse of the expected results and would counter this hypothesis [43]. After collapsing all the novel designer crossbreed dogs into one category, the current study identified designer crossbreed dogs overall with 1.34 times higher odds of acute diarrhoea compared to general crossbred dogs after accounting for age. Although this may represent a true predisposition, these results could also in part be attributable to an increased likelihood for owners of designer crossbreeds to seek veterinary care [44]. It is also possible that the increased odds shown here by designer crossbreeds overall is an example of ecological fallacy as a logical error from drawing conclusions about individuals based on data collected from a group [45]. This fallacy assumes that relationships that hold at one level of aggregation also hold at another level but it may be here that some individual novel designer crossbreeds are predisposed to acute diarrhoea but that this should not be interpreted as all designer crossbreeds having a similar predisposition. However, given that the designer crossbreeds Cavapoo and Cockapoo featured as two of the six breeds with predispositions in the current study, this could suggest a true predisposition at least in these two designer crosses. Based on data from a large survey of dog owners, the Cavapoo were previously reported with higher odds of acute diarrhoea compared to both progenitor breeds of Cavalier King Charles Spaniel and Poodle, while the Cockapoo were reported with higher odds than their poodle progenitor breed [46]. However, it is also possible that it is the poodle progenitor that might create a predisposition to acute diarrhoea in the Cavapoo and Cockapoo, given that the Miniature Poodle featured with the second highest odds ratio among the six predisposed breeds in the current study. Either way, it is important that owners and veterinary professionals are aware of disorder profiles for these novel designer crossbred breeds that are now becoming a major part of the national dog breed structure in the UK as well as many other countries [41,47].

The remaining three breeds identified with predisposition to acute diarrhoea in the current study were the Maltese Terrier, German Shepherd Dog and Yorkshire Terrier. Although Maltese have previously been described as predisposed to AHDS [36], German Shepherd Dogs have been reported as predisposed to chronic enteropathies and antibiotic-responsive diarrhoea [48,49] and Yorkshire Terriers to Yorkshire Terrier Enteropathy, the current study focused on uncomplicated acute diarrhoea overall and did not aim to determine whether these breeds were over-represented for particular acute diarrhoea subtypes. The current study also did not extract information on other factors such as diet, exercise and owner care-seeking behaviour that may have varied between breeds and therefore affected the risk of presentation with acute diarrhoea but that could be topics for future work [7,50].

The current results identified breeds with brachycephalic skull conformation as protected from acute diarrhoea while dolichocephalic dogs were predisposed, compared to dogs with mesocephalic skulls. Conformational anomalies commonly selected in brachycephaly breeds have previously been associated with predisposition to upper gastrointestinal disorders including hiatal hernia, pyloric stenosis and gastroesophageal reflux disease [51] but protection from acute diarrhoea has not been documented previously. As for the discussion on designer crossbreeds above, it is possible that these results for skull conformation represent ecological fallacy and it may be that the effects of certain individual over-represented breeds within each skull type may be driving these apparent skull type associations. Further studies are warranted to explore possible breed effects as greater awareness among owners of at-risk breeds could support targeted preventative measures.

Age was identified a major risk factor for acute diarrhoea in the current study, with both very young and very old dogs predisposed compared to middle aged dogs. Predisposition in young dogs aged under 3 years may be related to their typical behaviours such as scavenging that exposes them to dietary indiscretion or intoxication [50,52]. These scavenging tendencies may be curbed with effective dog training or mitigated by management strategies such as on-lead walks or the wearing of basket muzzles [53]. After dropping during middle-age, the incidence risk for acute diarrhoea rose sharply again in dogs aged 9 years and above. This could reflect an increasing contribution of extra-digestive aetiologies with increasing age or putative disturbances to gut health/function and the microbiota that are associated with the aging process [54,55]. Either way, owners should be made aware that a higher risk of acute diarrhoea occurrence in older dogs appears real and that greater diligence to monitor signs of digestive upset or even to consider prophylactic dietary strategies such as moving towards blander diets may be advisable [56].

Using ‘Big Data’ analysis to explain the effects of risk factors and to predict the probability of clinical outcomes has become an increasing active research area for data-scientists and veterinarians over recent decades, especially as veterinary care has become increasingly digitised to offer large samples of accessible digital footprints [57,58]. At a big picture level, the current study identifies acute diarrhoea as a highly predictable event for dogs, with an 8.18% annual incidence risk linked to a 95% confidence interval of 7.83–8.55 suggesting that a typical dog under veterinary care can be confidently stated to have a 1-in-12 probability of receiving active veterinary care for acute diarrhoea in any 12-month period. The results of the multivariable modelling also help predict what factors, all other things being equal, make the probability of receiving veterinary care even higher than 1-in-12, for example predicting higher than 1-in-12 risk for a typical Maltese dog or a dog aged 2 years. However, despite this high level of certainty at a population level, the current results also reveal high uncertainty for prediction of acute diarrhoea at an individual animal level, with the current final model explaining just 6.73% of the total variance in the data. This suggests that the overwhelming majority of variation (i.e., 93.27%) remains unexplained and may be attributable to other factors not captured in the current data or to random chance [59]. Other factors previously implicated as potential triggers of acute diarrhoea include viral infection [60], diet [61,62], diet change, including any overly-rapid dietary transition, dog behaviour [63], administration of medication, e.g., non-steroidal anti-inflammatory medication [64], antimicrobials [65] or proton-pump inhibitors [66] and exposure to toxins (e.g., insecticides).

Concurrent clinical signs were recorded with acute diarrhoea in over two thirds of cases in the current study. Concomitant vomiting in 44% of diarrhoeic dogs may be indicative of a more diffuse insult to the gastrointestinal tract (e.g., acute gastroenteritis or chronic enteropathy). However, maropitant use (24%) was barely half as frequent as the vomiting, suggesting that the vomiting was perceived as non-critical or perhaps self-resolving in many instances. Interestingly, antacids including proton pump inhibitors (PPIs) such as omeprazole and histamine H_2_ receptor antagonists such as cimetidine and ranitidine were also frequently used, particularly in dogs with concurrent vomiting despite a lack of evidence of any anti-emetic efficacy for these drugs (S3 File) [67]. Inappropriate use of omeprazole in veterinary teaching hospitals has been reported previously [68–70] prompting a call for improved prescriber education around the use of these classes of medication. The use of PPIs identified in the current study is even more questionable, given that acute diarrhoea is a commonly recognised adverse effect of that therapy [70,71] and has the potential to aggravate states of dysbiosis [72].

Antibiotic therapy for acute diarrhoea cases was frequently observed in the current study, with 38.2% of cases receiving at least one antibiotic treatment. This result appears much higher than 5.7% of consultations assigned a canine gastroenteritis diagnosis that received antibiotic therapy between 1st of January 2020 and 31st of December 2023 in Sweden [73]. However, some of this apparent difference may relate to the current study reporting antibiotic use per case whereas the Swedish study reported antibiotic use per consultation. Recent work applying novel methods to emulate a randomised controlled trial using observational veterinary clinical data has shown no clinical benefits from the use of antibiotic therapy for uncomplicated acute diarrhoea in dogs [16]. A European multidisciplinary panel (ENOVAT) has strongly recommended and with high certainty that antimicrobials should not be used to treat dogs with acute haemorrhagic or non-haemorrhagic diarrhoea that are showing moderate or milder disease [18]. The presence of blood within the diarrhoea appeared to be a strong driver of antibiotic prescription in the current study, in contradiction to current recommendations that antibiotics are not indicated to manage acute haemorrhagic diarrhoea syndrome in dogs [14,74]. As reported elsewhere [75–79], in the current study metronidazole was overwhelmingly the most popular antibiotic choice to manage acute diarrhoea. Unfortunately, the clinical rationale underlying this antibiotic selection cannot be determined from the current clinical records but both antibacterial and putative non-antibacterial properties have previously been cited as a justification to use metronidazole in dogs with diarrhoea [75]. Over 80% of acute diarrhoea cases in the current study were managed without performing further diagnostic tests. The presence of blood in the diarrhoea was not associated with increased use of laboratory testing but laboratory testing was associated with increased use of antibiotic therapy. Empiric use of antibiotics risks potentiating antimicrobial resistance and is discouraged for both acute [17] and chronic diarrhoea [80]. A greater understanding of the motivating factors underlying ongoing high antibiotic use despite widespread recommendations to the contrary could help improve future antimicrobial stewardship efforts. The current results showing that over 80% of cases do not receive a second veterinary visit suggest that the clinical outcomes following veterinary care are overwhelmingly good regardless of laboratory investigation or antibiotic therapy and that neither approach may be necessary if the primary aim of veterinary care is resolution of the clinical condition within a broader contextualised care approach [81]. Future work is planned to explore compliance with ENOVAT and BSAVA ‘Protect Me’ recommendations for antibiotic use in acute diarrhoea in dogs in the UK [18,20].

This study had several limitations that were largely linked to the secondary use of veterinary clinical data for research. Several of the variables had potentially high proportions of missing data which may reflect an absence of recording in the electronic record or an absence of assessment for the feature rather than genuine absence of clinical feature. For example, the documented pyrexia in 8.72% in acute diarrhoea cases may underestimate the true frequency because temperature may not have been measured in all cases or the result may not have been recorded for all cases where this was measured. It should be noted also, however, that some true events of hyperthermia due to stress or excitement may have been misclassified as pyrexia events in the clinical records and therefore led to over-estimation of proportional pyrexia [82]. It was not possible to evaluate the severity of the cases at presentation because this would require consistent reporting of clinical parameters (e.g., hydration status and temperature) across the clinics that shared data for the study, although this could have provided greater granularity to support or refute the value of various therapeutic interventions. The current study explored association between acute diarrhoea and certain breeds not just for genetic but also for other reasons. Environmental, epigenetic and socioeconomic factors may also be relevant if certain breeds presenting diarrhoea tend to be owned by less experienced or affluent dog owners, or who are inclined to feed their pets in particular ways (e.g., raw feeding) [52]. The perspective of the attending veterinarian at the first presentation with diarrhoea was adopted to decide whether each animal met the case definition for acute diarrhoea or not. This aimed to report on veterinary decision-making based on contemporaneous knowledge at that point rather than filtering out cases that may later become chronic. However, it should be noted that almost 1% of the acute diarrhoea cases were recorded with 3–5 acute diarrhoea events within the year of the study and over 7% had a history of clinical signs for one week or more prior to first veterinary presentation. It is possible that some of these cases were linked to an underlying chronic disease pathogenesis. The duration of antibiotic treatment for each case was not included in the current analysis.

Using data available within VetCompass in 2019 (pre-Covid pandemic) may limit generalisability of study findings to all UK primary care practices post-Covid). The study included a mix of private and corporate practices but with a predominance of the latter. This may have skewed the results towards the standards and policies of clinical management and care typical of these corporate practices in 2019. Of note, many UK corporates have introduced new guidelines around antibiotic use in the intervening years since 2019. Additionally, the study represents the demographic breed structure in dogs in 2019 [41]. The UK dog population may change over time with consequent changes in the frequency and nature of even the most common disorders at an overall population level. Increasing puppy numbers during 2020–2021 linked to the current study findings of increased acute diarrhoea risk in dogs under 3 years of age would predict a higher incidence risk for acute diarrhoea as the average age of the UK dog population drops [83].

### Conclusions

This study confirms acute diarrhoea as one of the most common clinical conditions in dogs managed under primary veterinary care, with 1-in-12 dogs diagnosed at least once each year. The breed predispositions identified suggest a genetic element to the condition. The clinical outcomes following veterinary care appear to be very positive, with over 80% of acute diarrhoea cases not requiring a second veterinary visit. However, high antibiotic use within veterinary clinical management against years of recommendation to the contrary raises concerns about unnecessary antibiotic therapy for this condition.

## Supporting information

S1 File Supplementary A: Clinical sign associated with the diarrhoea on the day of first veterinary presentation with acute diarrhoea during 2019 in dogs under primary veterinary care in the VetCompass™ Programme in the UK. N = 1835. (DOCX)

S2 File Supplementary B: Diagnostic testing performed on the first day of veterinary presentation with acute diarrhoea during 2019 in dogs under primary veterinary care in the VetCompass™ Programme in the UK. N = 1835. *cPLI canine pancreatic lipase immunoreactivity.
(DOCX)

S3 File Supplementary C: Treatments and clinical management used on the first day of veterinary presentation for acute diarrhoea during 2019 in dogs under primary veterinary care in the VetCompass™ Programme in the UK. N = 1835. (DOCX)

S4 File Supplementary D: Antibiotics and routes of administration prescribed on the first day of veterinary presentation with acute diarrhoea during 2019 in dogs under primary veterinary care in the VetCompass™ Programme in the UK. N = 701. (DOCX)

S5 File Supplementary E: Suspected trigger stated in the clinical records for acute diarrhoea events during 2019 in dogs under primary veterinary care in the VetCompass™ Programme in the UK. N = 1835. (DOCX)

## Abbreviations

AHDS: acute haemorrhagic diarrhoea syndrome
CI: confidence interval
EHR: electronic health record
IQR: interquartile range
KC: The Kennel Club
OR: odds ratio
PPI: proton pump inhibitors
RCT: randomised controlled trial
