# Supplementary material for: Epidemiology and clinical management of acute diarrhoea in dogs under primary veterinary care in the UK
Source: PLoS One. 2025 Jun 11;20(6):e0324203. doi: 10.1371/journal.pone.0324203 (PMC12156985; doi:10.1371/journal.pone.0324203)
Supplement: S1 File — (DOCX) [file pone.0324203.s001.docx]

Supplementary A: Clinical sign associated with the diarrhoea on the day of first veterinary presentation with acute diarrhoea during 2019 in dogs under primary veterinary care in the VetCompass™ Programme in the UK. N = 1835

| Clinical sign associated with the diarrhoea on the day of first veterinary presentation with acute diarrhoea | No. | % |
| --- | --- | --- |
| Vomiting | 812 | 44.25 |
| None recorded | 604 | 32.92 |
| Reduced appetite | 508 | 27.68 |
| Lethargy | 444 | 24.20 |
| Abdominal pain/discomfort | 246 | 13.41 |
| Pyrexia | 160 | 8.72 |
| Dehydration | 143 | 7.79 |
| Weight loss | 60 | 3.27 |
| Borborygmi | 50 | 2.72 |
| Flatulence | 45 | 2.45 |
| Pale mucous membranes | 43 | 2.34 |
| Neurological signs | 29 | 1.58 |
| Polyuria and polydipsia | 23 | 1.25 |
| Retching | 17 | 0.93 |
| Underweight | 16 | 0.87 |
| Distended abdomen | 14 | 0.76 |
| Red perineum | 13 | 0.71 |
| Scooting bottom along floor | 13 | 0.71 |
| Enlarged Lymph Nodes | 12 | 0.65 |
| Infected/ ruptured anal sac | 10 | 0.54 |
| Hypothermic | 10 | 0.54 |
| Coprophagia | 9 | 0.49 |
| Painful anus | 4 | 0.22 |
| Weak pulse | 3 | 0.16 |
| Epistaxis | 1 | 0.05 |
| Increased appetite | 1 | 0.05 |
| Anemia | 1 | 0.05 |
