# Supplementary material for: Epidemiology and clinical management of acute diarrhoea in dogs under primary veterinary care in the UK
Source: PLoS One. 2025 Jun 11;20(6):e0324203. doi: 10.1371/journal.pone.0324203 (PMC12156985; doi:10.1371/journal.pone.0324203)
Supplement: S2 File — *cPLI canine pancreatic lipase immunoreactivity. (DOCX) [file pone.0324203.s002.docx]

Supplementary B: Diagnostic testing performed on the first day of veterinary presentation with acute diarrhoea during 2019 in dogs under primary veterinary care in the VetCompass™ Programme in the UK. N = 1835. *cPLI canine pancreatic lipase immunoreactivity

| Diagnostic testing performed on the first day of veterinary presentation with acute diarrhoea | No. | % |
| --- | --- | --- |
| None testing recorded | 1511 | 82.34 |
| Haematology +/- biochemistry | 202 | 11.01 |
| Faecal analysis | 64 | 3.49 |
| Abdominal imaging | 64 | 3.49 |
| cPLI* SNAP Test (IDEXX UK) | 46 | 2.51 |
| Parvo SNAP test (IDEXX UK) | 13 | 0.71 |
| Urinalysis | 10 | 0.54 |
| Giardia SNAP test (IDEXX UK) | 5 | 0.27 |
| ACTH stimulation test | 2 | 0.11 |
| Clotting factors test | 1 | 0.05 |
