# Supplementary material for: Epidemiology and clinical management of acute diarrhoea in dogs under primary veterinary care in the UK
Source: PLoS One. 2025 Jun 11;20(6):e0324203. doi: 10.1371/journal.pone.0324203 (PMC12156985; doi:10.1371/journal.pone.0324203)
Supplement: S3 File — (DOCX) [file pone.0324203.s003.docx]

Supplementary C: Treatments and clinical management used on the first day of veterinary presentation for acute diarrhoea during 2019 in dogs under primary veterinary care in the VetCompass™ Programme in the UK. N = 1835.

| Treatments and clinical management used on the first day of veterinary presentation for acute diarrhoea | No. | % |
| --- | --- | --- |
| Probiotics | 1094 | 59.62 |
| Dietary management (e.g. starvation, bland diet, specialised Diets etc). | 807 | 43.98 |
| Antibiotics | 701 | 38.20 |
| Maropitant | 441 | 24.03 |
| De-wormer | 232 | 12.64 |
| Omeprazole | 181 | 9.86 |
| Ranitidine/cimetidine | 178 | 9.70 |
| Admitted for intravenous fluid therapy | 144 | 7.85 |
| No treatment documented | 135 | 7.36 |
| Hospitalisation | 132 | 7.19 |
| Opioids | 104 | 5.67 |
| Stop use of NSAIDs | 85 | 4.63 |
| Rehydration supplements | 65 | 3.54 |
| Systemic glucocorticoids | 63 | 3.43 |
| Paracetamol | 36 | 1.96 |
| Meloxicam | 30 | 1.63 |
| Sulfasalazine | 19 | 1.04 |
| Hyosine butylbromide | 17 | 0.93 |
| Vitamin B Injection | 12 | 0.65 |
| Metoclopramide | 12 | 0.65 |
| Stop/ reduce other current medication | 9 | 0.49 |
| Gut motility drugs | 7 | 0.38 |
| Admitted for subcutaneous fluid therapy | 7 | 0.38 |
| Activated charcoal | 7 | 0.38 |
| Prednisolone Tablets | 7 | 0.38 |
| Emetics | 5 | 0.27 |
| Exploratory laparotomy | 4 | 0.22 |
| Anal sacs emptied | 4 | 0.22 |
| Robenacoxib | 3 | 0.16 |
| Sucralfate | 3 | 0.16 |
| Gabapentin | 2 | 0.11 |
| Betamethasone valerate and fusidic acid skin gel | 2 | 0.11 |
| Loperamide | 2 | 0.11 |
| Psyllium Hhsk | 2 | 0.11 |
| Nasogastric tubing | 2 | 0.11 |
| Anti-histamines | 1 | 0.05 |
