# Supplementary material for: Epidemiology and clinical management of acute diarrhoea in dogs under primary veterinary care in the UK
Source: PLoS One. 2025 Jun 11;20(6):e0324203. doi: 10.1371/journal.pone.0324203 (PMC12156985; doi:10.1371/journal.pone.0324203)
Supplement: S4 File — (DOCX) [file pone.0324203.s004.docx]

Supplementary D: Antibiotics and routes of administration prescribed on the first day of veterinary presentation with acute diarrhoea during 2019 in dogs under primary veterinary care in the VetCompass™ Programme in the UK. N = 701

| Antibiotics and routes of administration prescribed on the first day of veterinary presentation with acute diarrhoea | No. | % |
| --- | --- | --- |
| Metronidazole - oral | 457 | 65.19 |
| Amoxicillin - parenteral | 159 | 22.68 |
| Amoxicillin - oral | 116 | 16.55 |
| Metronidazole - parenteral | 67 | 9.56 |
| Metronidazole and spiramycin - oral | 8 | 1.14 |
| Cefovecin - parenteral | 3 | 0.43 |
| Clindamycin - oral | 3 | 0.43 |
| Erythromycin - oral | 2 | 0.29 |
| Oxytetracycline - oral | 1 | 0.14 |
| Tylosin - oral | 1 | 0.14 |
| Cephalexin - oral | 1 | 0.14 |
| Enrofloxacin - oral | 1 | 0.14 |
| Lincosamide - parenteral | 1 | 0.14 |
