# Supplementary material for: Epidemiology and clinical management of acute diarrhoea in dogs under primary veterinary care in the UK
Source: PLoS One. 2025 Jun 11;20(6):e0324203. doi: 10.1371/journal.pone.0324203 (PMC12156985; doi:10.1371/journal.pone.0324203)
Supplement: S5 File — (DOCX) [file pone.0324203.s005.docx]

Supplementary E: Suspected trigger stated in the clinical records for acute diarrhoea events during 2019 in dogs under primary veterinary care in the VetCompass™ Programme in the UK. N = 1835.

| Recorded suspected trigger or explanation for acute diarrhoea event | No. | % |
| --- | --- | --- |
| None recorded | 736 | 40.11 |
| Scavenging/dietary indiscretion | 175 | 9.54 |
| Gastroenteritis | 149 | 8.12 |
| Colitis | 85 | 4.63 |
| Reaction to NSAID therapy | 85 | 4.63 |
| Dietary change | 84 | 4.58 |
| Parasitic | 78 | 4.25 |
| Haemorrhagic gastroenteritis | 66 | 3.60 |
| Multiple differentials | 57 | 3.11 |
| Pancreatitis | 43 | 2.34 |
| Infection | 33 | 1.80 |
| Stress | 33 | 1.80 |
| Raw food | 32 | 1.74 |
| Sensitive stomach | 29 | 1.58 |
| Adverse drug reaction | 26 | 1.42 |
| Neoplasia | 25 | 1.36 |
| Toxic insult | 16 | 0.87 |
| Ingested foreign dody | 13 | 0.71 |
| Inflammatory bowel disease | 12 | 0.65 |
| Anal sac infection | 11 | 0.60 |
| Kidney disease | 11 | 0.60 |
| Pyometra | 9 | 0.49 |
| Addison’s disease | 5 | 0.27 |
| Old age | 4 | 0.22 |
| Campylobacteriosis | 3 | 0.16 |
| Intussusception | 2 | 0.11 |
| Phantom pregnancy | 2 | 0.11 |
| Protein losing enteropathy | 2 | 0.11 |
| Acute haemorrhagic diarrhoea syndrome | 1 | 0.05 |
| Anaphylaxis/allergic reaction | 1 | 0.05 |
| Cholangitis | 1 | 0.05 |
| Cushing’s disease | 1 | 0.05 |
| Dysbiosis | 1 | 0.05 |
| Leptospirosis | 1 | 0.05 |
| Liver pathology | 1 | 0.05 |
| Parvovirus | 1 | 0.05 |
| Vestibular disease | 1 | 0.05 |
